# Carry-over effects in GAG therapy efficacy trial solution for bladder pain syndrome/interstitial cystitis (GETSBI study): an interim analysis

**DOI:** 10.1136/bmjopen-2024-092757

**Published:** 2025-06-05

**Authors:** Charlotte van Ginkel, J M M Groenewoud, Thomas J Hoogeboom, John Heesakkers, Frank Martens, Dick Janssen

**Affiliations:** 1 Department of Urology, Radboud University Medical Center, Nijmegen, The Netherlands; 2 Radboudumc, Nijmegen, The Netherlands; 3 Radboud Institute for Health Sciences, IQ healthcare, Radboud University Medical Center, Nijmegen, The Netherlands; 4 Department of Urology, Maastricht UMC+, Maastricht, The Netherlands

**Keywords:** Interstitial cystitis, Bladder disorders, STATISTICS & RESEARCH METHODS, THERAPEUTICS, Randomized Controlled Trial

## Abstract

**Objectives:**

The double blind, multicentre, randomised, placebo-controlled GAG-therapy Efficacy Trial Solution for Bladder pain syndrome/Interstitial cystitis (GETSBI) study aims to evaluate the efficacy of intravesical glycosaminoglycans therapy with hyaluronic acid and chondroitin sulfate in symptomatic bladder pain syndrome/interstitial cystitis (BPS/IC) patients with Hunner lesions. This trial encompasses multiple methodologies, including a standard randomised controlled trial (RCT), a cross-over trial and an N-of-1 trial. An N-of-1 trial is a multiple crossover trial, usually randomised and often blinded, conducted in a single patient (1). The N-of-1 methodology is, however, only valid under the condition that there is no carry-over effect, meaning a carry-over of effect from an a-priori intervention period into the placebo period. Therefore, it is important to examine any potential carry-over effects to determine the validity of the study protocol concerning the N-of-1 trial part and thereby justifying recruitment.

**Design:**

Interim analysis for potential carry-over effects.

**Setting:**

Secondary care, 21 participants.

**Participants:**

21 participants, participants concluded part one from the GETSBI study at time of this analysis (October 2023).

**Outcome measure:**

The primary outcome of the study is the change from baseline in pain intensity, measured by visual analogue scale (VAS) pain. To assess for carry-over effects, the placebo responses on VAS pain were compared between groups with (n=10) and without (n=11) potential carry-over effects. The threshold for a clinically relevant carry-over effect was set at a difference on VAS pain >0.50 points. Data were analysed using descriptive statistics, T-tests, effect sizes and 95% CI. Statistical significance was set at α=0.05.

**Results:**

The mean baseline VAS pain did not differ (p=0.12) between group A (n=10, VAS 7.52, SD=0.52) and group B (n=11, VAS 6.02, SD=2.47). The mean placebo responses on VAS pain for groups A and B were 0.97 (SD=1.85) and 1.47 (SD=1.81), respectively. The mean carry-over effect was 0.50 (SD=1.83), which was not statistically significant with a 95% CI of −1.17 to 2.17 and p=0.5369.

**Conclusions:**

This interim analysis shows that an N-of-1 trial probably will be feasible for evaluating non-curative treatment efficacy in chronic disease using only half the patients as are required for a classic RCT. Future analysis will provide a direct comparison of outcomes between the RCT, crossover and the N-of-1 part for a complete evaluation.

**Trial registration number:**

ClinicalTrials.gov, NCT05518864 (GETSBI study).

**STRENGTHS AND LIMITATIONS OF THIS STUDY**

- The simple design of the interim analysis enhances the transparency of the research process.
- The timing of this interim analysis is mandated by regulatory authorities as a prerequisite for the continuation of the study.
- Evaluating carry-over effects using a threshold value presents an intrinsic limitation due to the difficulty in precisely determining an appropriate threshold.
- One risk associated with an interim analysis is that the effect sizes obtained from such early analyses are at risk of being inflated.
- Only the primary outcome of the GAG-therapy Efficacy Trial Solution for Bladder pain syndrome/Interstitial cystitis study is considered in this interim analysis.

## Introduction

 The double-blind, multicentre, randomised, placebo-controlled GAG-therapy Efficacy Trial Solution for Bladder pain syndrome/Interstitial cystitis (GETSBI) study, started recruiting patients at the end of 2021 (ClinicalTrials.gov identifier (NCT number): NCT05518864). The study investigates the efficacy of intravesical glycosaminoglycans (GAG) therapy with hyaluronic acid (HA) and chondroitin sulfate (CS) in symptomatic bladder pain syndrome/interstitial cystitis (BPS/IC) patients with Hunner lesions. This study is part of the ‘conditional reimbursement’ programme in the Netherlands and has received approval from the Dutch Ministry of Healthcare. The purpose of the study is to assess the efficacy and thereby facilitate the reinstatement of reimbursement for this particular GAG therapy in this specific patient group.

In March 2023, the study protocol of the study was published in BMJ Open, serving as a reference for specific protocol details.^1^ The GETSBI study is a multi-method study, starting as a standard randomised controlled trial (RCT), continuing into a cross-over and an N-of-1 trial. The latter is a multi-crossover design with multiple cycles of treatment and placebo. Both a crossover and an N-of-1 design evaluate intervention and placebo within an individual participant. This is of additional value when the primary outcome measure is a subjective measurement like pain or urinary urgency. With Bayesian analyses, one can combine or aggregate the individual results of individual N-of-1 trials and obtain level one evidence.^2 3^ Crossover studies and especially aggregated N-of-1 trials require far fewer patients for adequate power, as intervention and placebo are evaluated in a single person. This implies that overall, you need fewer patients when a study implements increasing numbers of intervention/placebo periods per patient. These methods are limited to chronic, non-curable diseases and their treatments. BPS/IC is a rare disease. In the Netherlands, there are approximately 1800 patients who are being treated for BPS/IC.^4^ The BPS/IC subtype with Hunner lesions accounts for approximately 10–55% of all BPS/IC patients and is therefore a rare subtype of an already rare disease.^5^,^10^ Incorporating a cross-over and an N-of-1 trial in the study design allows examination of whether effective intervention trialling can also be performed with lower inclusion numbers compared with classic RCT methodology. This is beneficial for rare diseases such as BPS/IC, where inclusion rates frequently fall short, as well as reduce participant burden. The N-of-1 trial methodology is only valid under the condition that there is no carry-over effect. This means an effect from an a-priori intervention period going over into the placebo period directly thereafter.^11^ Therefore, the aim of this study is to examine any potential carry-over effects to evaluate the validity of the N-of-trial and ensure that continuing recruitment and participant exposure under the current protocol is justifiable and has value.

## Methods

This interim analysis is performed on data from the double blind, multi centre, randomised, placebo-controlled trial named GETSBI study (ClinicalTrials.gov identifier (NCT number): NCT05518864).^1^ This interim analysis aims to evaluate potential carry-over effects to assess the integrity of the N-of-1 trial design and to ensure that ongoing recruitment and participant exposure under the current protocol are both scientifically warranted and ethically sound.

### Study design

**Figure 1:**
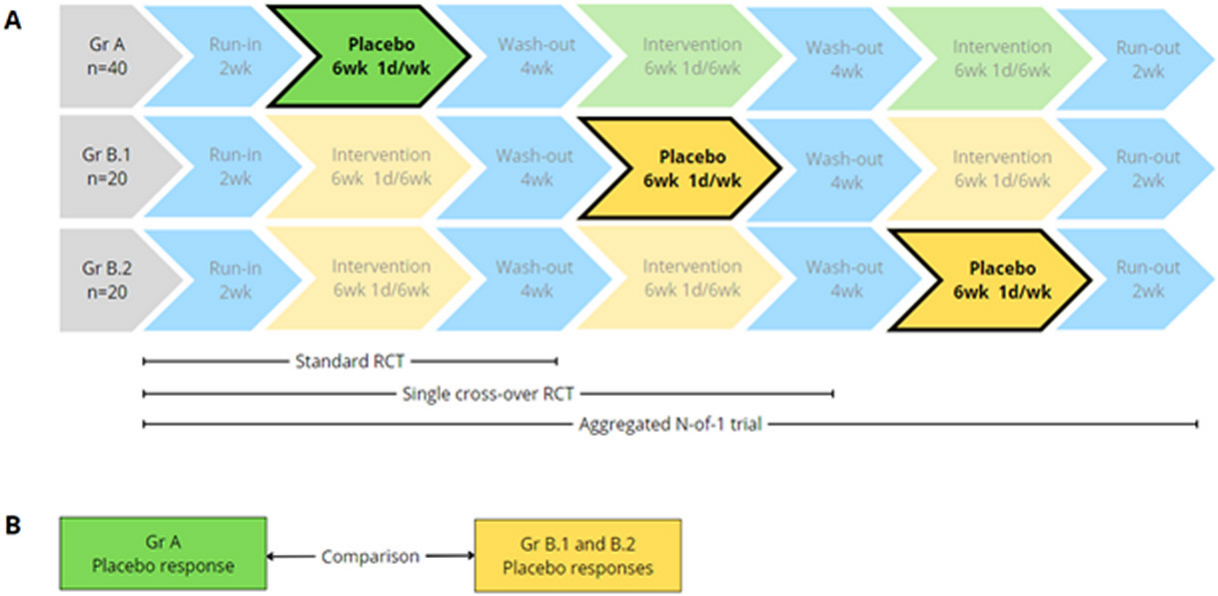
Trial flowchart.

The GETSBI study is a double blind, multicentre, randomised, placebo-controlled trial, encompassing a classic RCT, cross-over trial and an N-of-1 trial. Patients undergo three treatment periods of six consecutive weeks with weekly bladder instillations, with an intervention: placebo ratio of 2:1 (ClinicalTrials.gov identifier (NCT number): NCT05518864).^1^ Efficacy of the primary outcome measurement is determined in weeks 5 and 6 of each period. Each participant is randomised in one of the three parallel arms determining the position of the placebo period (period one, two or three). Group A starts with the placebo in period one and is unaffected by any carry-over effect. Group B.1 and B.2 have, prior to their placebo period, one or two periods with intervention. These patients are potentially affected by carry-over effects. To prevent carry-over effects, there are four wash-out weeks between periods, which corresponds to a total of 8 weeks between the last intervention of the prior period and the measurements of the primary outcome measurement in the placebo period for groups B.1 and B.2. As seen in figure 1, the first 6 weeks (first treatment period) embodies the RCT part, with an equal distribution of placebo and intervention. Patients then continue with periods two and three and are randomised for intervention or placebo.

The last 6 months of the study is an open, non-blinded prospective part to evaluate the long-term efficacy where patients are given 6 monthly bladder instillations with the active product. This phase does not fall in the scope of this article.

### Eligibility criteria

Adult symptomatic BPS/IC patients with cystoscopically confirmed Hunner lesions are eligible for inclusion. For details regarding inclusion and exclusion criteria, see protocol (ClinicalTrials.gov identifier (NCT number): NCT05518864).^1^

### Intervention

The intervention with the active product is HA 1.6% and CS 2% (Ialuril prefill, IBSA, Goodlife). This product is registered as a medical device (CE 0477) and is instilled into the bladder with a disposable urinary catheter by an experienced nurse. For placebo, artificial tears are used, since it is an inert gel (non-irritating and hypo-allergenic) that accurately mimics the investigational product (viscosity). This will also be instilled into the bladder with a disposable urinary catheter in blinded syringes.

### Study outcomes

The primary outcome of the study is the intensity of maximal pain for 3 days measured by a visual analogue scale (VAS pain, range 0–10). Zero implies no pain, and ten is the worst pain the participant has ever experienced. Efficacy of the GAG therapy is defined as an improvement of two points on the VAS pain score after intervention compared with baseline (before any treatment). In weeks 5 and 6 of each treatment period, the efficacy measurements are done (four measurements in 2 weeks). In the case of the placebo period, these measurements account for the placebo effect.

### Power calculation

In short, the study will be powered (>80%) for a standard RCT with 80 patients (including 22 patients for potential dropouts). For the N-of-trial, the required number of inclusions is 38 patients (including 10 patients for dropouts).

### Study protocol and registration

For further elaboration on study outcomes and power calculations, as well as additional information regarding recruitment and randomisation, blinding and statistical analysis, the protocol publication in BMJ Open can be consulted. Also, a complete overview of the study protocol is given in the registration on ClinicalTrials.gov (identifier number (NCT number): NCT05518864).

#### Analysis of potential carry-over effects

The N-of-1 trial method is only valid under the condition that there is no carry-over effect.^11^ Half of the patients (group B.1 and B.2) switch from receiving the intervention initially to receiving a placebo later, which could potentially introduce carry-over effects from the intervention phase to the placebo phase. Therefore, it is crucial to investigate these potential carry-over effects to ascertain the validity of the N-of-1 trial. To evaluate the presence of carry-over effects, the placebo responses regarding VAS pain were compared between Group B (comprising both B.1 and B.2) and Group A, where the latter group is not influenced by carry-over effects (see figure 1). A predetermined threshold for clinically relevant carry-over effect was set at a VAS pain difference >0.50 points between the two groups. This threshold was pre-determined by the stakeholders that includes a patient panel who engaged in designing the study protocol.

### Statistical analysis

Data was analysed using descriptive statistics, T-tests, effect sizes and 95% CI. Statistical significance was set at α=0.05. The carry-over effect was assumed to be one-sided, indicating that if present, pain VAS measurements in group B would be significantly lower compared with those in group A. The analysis was performed by a statistician who was blinded for group allocation.

### Patient and public involvement

Patients and/or the public were involved in the design for the main study. They were informed about the continuation of the study after this interim analysis. This was done through the Patient Association Interstitial Cystitis.

## Results

### Overall recruiting numbers

At the time of the interim analysis, a total of 35 patients had been recruited for the study over a period of 20 months. There were four dropouts (11%), with one occurring during phase 1 of the study and the remaining three after phase 1. Of the 35 patients, 26 completed phase 1 of the study. Five patients did not respond to the questionnaire within a reliable period (within 3 days) and were excluded from the analysis. Therefore, 21 patients were enrolled in the interim analysis.

### Carry-over analysis

Among these 21 patients, 10 patients initiated the study with the placebo period (group A), thereby eliminating any possible chance for carry-over effect. While the other 11 patients began with an intervention period before transitioning to the placebo period (group B), these patients are susceptible to potential carry-over effects from the intervention in their placebo period.

The mean baseline VAS pain between groups showed no statistically significant difference, respectively, 7.52 (SD=1.52) for group A and 6.02 (SD=2.47) for group B (p=0.1157). The mean placebo responses on VAS pain for group A and B were respectively 0.97 (SD=1.85) and 1.47 (SD=1.81). The mean difference (95% CI) in placebo response (carry-over effect) was 0.50 (−1.17 to 2.17) was not statistically significant (p=0.54). Table 1 provides a summary of the data.

**Table 1:** Comparison of visual analogue scale pain scores of group A (unaffected by potential carry-over effect) and group B (affected by potential carry-over effect)

	VAS pain baseline		VAS pain placebo period		Placebo response (difference in VAS)*	
	Mean	SD (95% CI)	Mean	SD (95% CI)	Mean	SD(95% CI)
Group A (n=10)*Unaffected by potential carry-over effect*	7.52	1.52 (6.43 to 8.60)	6.55	1.54 (5.45 to 7.65)	−0.97	1.85 (−2.29 to 0.35)
Group B (n=11)*Affected by potential carry-over effect*	6.02	2.47 (4.36 to 7.68)	4.55	1.82 (3.33 to 5.78)	−1.47	1.81 (−2.69 to 0.25)
			*Mean*	SD (95% CI*)*		
Carry-over effect (difference in placebo response between group A and group B)			0.50	1.83 (−1.17 to 2.17)		

* Difference in VAS pain between VAS pain baseline and VAS pain efficacy in placebo period.

VAS, visual analogue scale.

## DISCUSSION

The purpose of this interim analysis was to identify any potential carry-over effects, to validate the cross-over design and to ensure that continuing recruitment and exposing patients to current protocol is justifiable and has value. The measured carry-over effect was 0.50 points on the VAS pain (95% CI −1.17 to 2.17), which did not reach statistical significance and was on the verge of clinical significance.

The threshold for a clinically relevant carry-over effect was arbitrarily established and based on the efficacy threshold within the GETSBI study. This threshold for efficacy in the GETSBI study means that efficacy is achieved when compared with placebo. There is an improvement of at least two points on the VAS pain score of the maximal pain in the last 3 days. The stakeholders discussed and agreed on that a carry-over effect exceeding 25% of the minimal efficacy threshold could potentially influence the analysis. This translates to >0.50 points on VAS pain score. Evaluating carry-over effects using a threshold value presents an intrinsic limitation due to the difficulty in precisely determining an appropriate threshold. Dichotomising data into ‘significant’ or ‘non-significant’ categories, similar to the conventional dichotomisation of data based on p values greater or smaller than 0.05, leads to information loss.^12^ Hence, one might question the significance of values near the threshold, such as whether 0.49 truly differs from 0.51. Pragmatic considerations often necessitate binary (yes or no) decisions within studies. It is important to acknowledge that thresholds do not guarantee the correctness of decisions. Threshold values can aid in forming judgement about the data and reduce ambiguity, but researchers should bear in mind the contextual factors to derive scientific inferences.^13^ Therefore, in this study, our measured carry-over effect approached the threshold closely, complicating the assessment of its potential impact on the final analysis. While we cannot definitely determine the impact yet, the threshold was not exceeded. Given the nature and significance of this study, meaning the potential of N-of-1 trials within this research field, as well as their application to other chronic diseases in the field of urology, such as other forms of inflammatory cystitis, it is both valid and appropriate to continue recruitment for this study.

Only statistical testing has been conducted for this study. Perhaps a different view might have emerged through the more nuanced approach of visual inspection.^14^ With visual inspection, independent experts from diverse backgrounds are blinded and queried to ascertain their expectations regarding carry-over effects and whether they perceive them as problematic. This approach aims to prevent the risk posed by strict cut-off values (thresholds) to result in harsh conclusions based on the smallest changes.

Another challenge is that this analysis was performed on only 21 patients, leading to the possibility that effect sizes derived from such early analyses are often inflated.^15^,^18^ This interim analysis was mandated at that point in time by authorities because the GETSBI study is part of the Dutch government’s ‘temporary reimbursement’ programme. Inflation is expected when both the outcome must meet a certain threshold of statistical significance and the study is underpowered. This is a common issue during interim analyses. Therefore, it is crucial to recognise this potential inflation when conducting interim analysis. Many studies are stopped prematurely due to positive results in interim analyses, which, with adequate power, might not have reached significance. The interim analysis for the GETSBI study focused solely on the carry-over effect during the placebo period. The primary efficacy outcome has not yet been analysed, ensuring that these outcomes will be assessed with appropriate power and without bias.

The study design of the GETSBI study offers valuable insights into methodological considerations for studying BPS/IC. Given that BPS/IC is a rare disease, studies often struggle to include enough patients, resulting in underpowered outcomes and low level of evidence recommendations in guidelines. By incorporating the N-of-1 methodology, patient burden is reduced, because first, fewer patients are needed and second, patients receive treatments beyond just placebo. This approach makes the study more appealing to patients and can improve inclusion rates. The GETSBI study allows for a comparison between the outcomes of a traditional RCT and the N-of-1 trial methodology, providing a comprehensive evaluation of this methodology’s first use in clinical urologic research.

## Conclusion

The carry-over effect identified in this interim analysis approaches the a priori threshold for clinically relevant carry-over effects, making it challenging to assess the potential impact on the final analysis. Although we cannot definitively state the impact at this stage, the threshold was not exceeded. Given the nature and significance of the study, continuing recruitment and proceeding with the N-of-1 part are valid. Overall, the study’s design and interim analysis provide valuable insights into the methodological considerations and challenges of studying BPS/IC and other rare diseases, highlighting the potential of N-of-1 trials in this research field.

## Data Availability

Data are available upon reasonable request.
