# Supplementary material for: Carry-over effects in GAG therapy efficacy trial solution for bladder pain syndrome/interstitial cystitis (GETSBI study): an interim analysis
Source: BMJ Open. 2025 Jun 5;15(6):e092757. doi: 10.1136/bmjopen-2024-092757 (PMC12142162; doi:10.1136/bmjopen-2024-092757)
Supplement: Supplementary file 1 [file bmjopen-2024-092757.reviewer_comments.pdf]

PEER REVIEW HISTORY

BMJ Open publishes all reviews undertaken for accepted manuscripts. Reviewers are asked to complete a checklist review form and are provided with free text boxes to elaborate on their assessment. These free text comments are reproduced below.

ARTICLE DETAILS

Title (Provisional)

Carry-over effects in GAG therapy Efficacy Trial Solution for Bladder pain syndrome/Interstitial cystitis (GETSBI study): an interim analysis

Authors

van Ginkel, Charlotte; Groenewoud, J.M.M.; Hoogeboom, Thomas J; Heesakkers, John; Martens, Frank; Janssen, Dick

---

VERSION 1 - REVIEW

**Reviewer**	**1**
**Name**	**Karaburun, Murat Can**
**Affiliation**	**Ankara Etlik City Hospital**
**Date**	**04-Jan-2025**
**COI**	**None**

I would like to thank the authors for this meticulous study. the purpose of the study and the results are clearly presented.

As stated in the text, unfortunately, studies to be conducted in such rare chronic diseases cannot reach a sufficient level of evidence. In urology, the BPS/IC patient group is one of the leading ones. I think this study can be a model for researchers in this context.

**Reviewer**	**2**
**Name**	**Birch, Brian**
**Affiliation**	**Southampton University Hospitals NHS Trust, Urology**
**Date**	**23-Feb-2025**
**COI**	**Possibly, in that I am employed in an advisory capacity (paid) to Consilient healthcare who are involved in the marketing of Elmiron for the treatment of Interstitial cystitis/BPS.in the UK**

This is an interesting paper examining the problems of study design when researching chronic conditions which are rare. The difficulty is in deciding what constitutes rare.

Estimates for this vary by author and country, with prevalence for IC/BPS in the US being estimated 0.87%, which would not necessarily be considered rare, and higher values have been reported.

Furthermore there are only 21 patients being considered for a carry-over effect, which is a small number, and as such the results are somewhat premature. I feel that more time is needed to allow any firm conclusions can been drawn regarding this.

Only one clinical feature was chosen to measure carry over effects and that was pain. Whilst this is undoubtedly important no other component affecting QoL was assessed, including those relating to urinary symptoms such as frequency, urgency and nocturia. Also, why were other assessments for IC/BPS not used for grading severity of disease such as the O'Leary Sant pain and voiding indices?

Thus, on balance, I feel the results are not mature enough yet for publication but would be happy to review this paper again if submitted at a future date.

**Reviewer**	**3**
**Name**	**Blasco, Pedro**
**Affiliation**	**Department of Urology, Hospital Universitario de Valme, Urology**
**Date**	**12-Mar-2025**
**COI**	**None**

After reviewing the article, I have one concern: Since this study is part of a larger research project, I'm unsure if it makes sense to publish a separate paper focusing on the crossover effect, as this analysis could be included in the final report.

Additionally, while this paper presents specific results regarding one aspect of a RCT, I believe it would be helpful to clarify why long-term follow-up is assessed after only six weekly instillations, especially given the existence of long-term treatment schemes. I would also appreciate a discussion on the various long-term treatment strategies.

VERSION 1 - AUTHOR RESPONSE

Reviewer: 1
Dr. Murat Can Karaburun, Ankara Etlik City Hospital

Comments to the Author:
I would like to thank the authors for this meticulous study. the purpose of the study and the results are clearly presented.
As stated in the text, unfortunately, studies to be conducted in such rare chronic diseases cannot reach a sufficient level of evidence. In urology, the BPS/IC patient group is one of the leading ones. I think this study can be a model for researchers in this context.
Thank you for your kind words. We hope with this study to enrich the research field concerning BPS/IC.

Reviewer: 2
Mr. Brian Birch, Southampton University Hospitals NHS Trust
Comments to the Author:
This is an interesting paper examining the problems of study design when researching chronic conditions which are rare. The difficulty is in deciding what constitutes rare. Estimates for this vary by author and country, with prevalence for IC/BPS in the US being estimated 0.87%, which would not necessarily be considered rare, and higher values have been reported.
Thank you for your comment. In the Netherlands, where this study was conducted, the prevalence of BPS/IC is estimated to be approximately 8-16 per 100.000 persons, expolarated to round 1800 patients in 2022 in the Netherlands, which is considered rare. Also IC/BPS is considered a rare disease by the European ERN (European Reference Network). The heterogeneity of BPS/IC adds further complexity to the disease, but we believe it also offers important opportunities to understand differences in treatment efficacy. However, sub-selection of patient groups inevitably reduces the number of eligible participants. In addition to the challenges related to the rarity of the condition, our study model may help to reduce patient burden and financial costs, both of which are important considerations that can limit the feasibility of clinical research.

Furthermore there are only 21 patients being considered for a carry-over effect, which is a small number, and as such the results are somewhat premature. I feel that more time is needed to allow any firm conclusions can been drawn regarding this.
We will comment on this below.

Only one clinical feature was chosen to measure carry over effects and that was pain. Whilst this is undoubtedly important no other component affecting QoL was assessed, including those relating to urinary symptoms such as frequency, urgency and nocturia. Also, why were other assessments for IC/BPS not used for grading severity of disease such as the O'Leary Sant pain and voiding indices?
We fully agree that multiple factors contribute to quality of life, and these aspects, including those you have highlighted, are incorporated into the main study design. The study is powered on VAS pain as primary outcome measurement, therefore this is also the necessary outcome measurement for the carry-over analysis.

Thus, on balance, I feel the results are not mature enough yet for publication but would be happy to review this paper again if submitted at a future date.
This interim analysis was commissioned by the Dutch Government as a decision point (go/no-go) for the continuation of the study. Consequently, the analysis was performed on the first 21 participants enrolled. We believe that the publication of this interim analysis enhances transparency within the context of this unique study. With this interim analysis we try to validate the aggregated N-of-1 trial methodology in this study, which we already demonstrate with only the first 21 first participants. This carry-over analysis will be repeated

in the final analysis.

Reviewer: 3
Dr. Pedro Blasco, Department of Urology, Hospital Universitario de Valme
Comments to the Author:
After reviewing the article, I have one concern: Since this study is part of a larger research project, I'm unsure if it makes sense to publish a separate paper focusing on the crossover effect, as this analysis could be included in the final report.
This interim analysis was commissioned by the Dutch Government as a decision point (go/no-go) for the continuation of the study. Consequently, the analysis was performed on the first 21 participants enrolled. We believe that publication of this interim analysis enhances transparency within the context of this unique study and the broader process of its execution. With this interim analysis we try to validate the aggregated N-of-1 trial methodology in this study, which we already demonstrate with only the first 21 first participants. We hope that it may also encourage other research groups to adopt similar study methodologies.

Additionally, while this paper presents specific results regarding one aspect of a RCT, I believe it would be helpful to clarify why long-term follow-up is assessed after only six weekly instillations, especially given the existence of long-term treatment schemes. I would also appreciate a discussion on the various long-term treatment strategies.
Long-term follow-up is assessed after six months of monthly treatments in the second, open-label phase of the study. The GETSBI study initially begins as a blinded, placebo-controlled trial to evaluate short-term efficacy, followed by an open-label phase. We consider this duration sufficient to make a meaningful assessment of the efficacy of GAG therapy, while a longer treatment period means increased patient burden and prolonging of the overall study duration.

*** ***

COI statements:

Reviewer: 1
If you have selected ‘Yes’ above, please provide details of any competing interests.: None.

Reviewer: 2
If you have selected ‘Yes’ above, please provide details of any competing interests.: Possibly, in that I am employed in an advisory capacity (paid) to Consilient healthcare who are involved in the marketing of Elmiron for the treatment of Interstitial cystitis/BPS in the UK

Reviewer: 3
If you have selected ‘Yes’ above, please provide details of any competing interests.: Not applicable.
